# Mesenchymal stroma/stem‐like cells of GARP knockdown inhibits cell proliferation and invasion of mouse colon cancer cells (MC38) through exosomes

**DOI:** 10.1111/jcmm.16008

**Published:** 2020-11-05

**Authors:** Haizhou Xing, Chunyan Liang, Xintong Xu, Hui Sun, Xiaojun Ma, Zhongxing Jiang

**Affiliations:** ^1^ Department of Hematology the First Affiliated Hospital of Zhengzhou University Zhengzhou China

**Keywords:** cancer therapy, cell proliferation, exosome, GARP, IL‐6, invasion, JAK1/STAT3 pathway, MC38 cells, migration, MSC

## Abstract

Mesenchymal stroma/stem‐like cells (MSCs) have antitumour activity, and MSC‐derived exosomes play a role in the growth, metastasis and invasion of tumour cells. Additionally, glycoprotein A repetition predominant (GARP) promotes oncogenesis in breast cancer. Therefore, GARP is speculated to be a target gene for cancer therapy. We aimed to explore the therapy role of MSC‐derived exosomes targeting GARP in mouse colon cancer cell MC38. We successfully established a GARP knockdown system using three kinds of siRNA‐GARP in MSC cells. Exosomes were isolated from MSC and siGARP‐MSC cells, and verified by the exosome surface protein markers CD9, CD63 and CD81. GARP expression was significantly decreased in siGARP‐MSC exosomes compared with that of MSC exosomes. We found that siGARP‐MSC exosomes inhibited cell proliferation, migration and invasion of MC38 cells, using CCK‐8, colony formation, wound‐healing and Transwell invasion assays. Furthermore, siGARP‐MSC exosomes impeded IL‐6 secretion and partly inactivated JAK1/STAT3 pathway, measured using ELISA and RT‐qPCR. In conclusion, MSC‐derived exosomes targeting GARP are a potential strategy for cancer therapy.

## 1 INTRODUCTION

Exosomes are cup‐shaped extracellular small nanovesicles, with a diameter ranging from 30 to 100 nm, and composed of a phospholipid bilayer containing membrane proteins.^1^ Exosomes are released into the extracellular compartment that comprises a large panel of proteins, mRNAs and regulatory microRNAs, directly shuttling the bioactive molecules among recipient cells.^2^ The role of exosomes has been figured out in cancer, contributing to tumour growth, angiogenesis, escaping from the immune response, causing tumour cell migration and stimulating invasion of normal cells.^3^ Most cells release exosomes, including cancer cells and populations that can associate with tumour tissue, such as heterogeneous MSCs.^4^ MSCs are believed to have antitumour effects and mediate their therapeutic functions in a paracrine, rather than a cellular, manner. Growing evidence suggests that MSC‐derived exosomes could transfer proteins and RNAs to recipient cells and exert several effects on the growth, metastasis and invasion of various tumour cells.^5^

GARP, also known as leucine‐rich repeat containing 32 (LRRC32), has been shown to serve as a receptor for latent TGF‐β1(LTGF‐β1), and activate CD4^+^ Foxp3^+^ Tregs and megakaryocytes/platelets.^6^ GARP is commonly expressed on induced regulatory T cells (Tregs), platelets and cancer cells, and plays a vital role in the maturation of LTGF‐β1, resulting in cancer progression.^7^, ^8^ Furthermore, GARP has been demonstrated to be expressed on the surface of mouse and human MSCs, colocalizing with membrane‐bound TGF‐β1.^9^ GARP gene was first described in human breast cancer, and GARP expression was found in only a few primary solid cancers, such as breast, colon, papillary thyroid and lung cancers.^10^, ^11^ However, the research of GARP in the mouse colon cancer was rare.

Here, we reported that a GARP knockdown system was constructed in the MSCs and exosomes were extracted from MSCs and MSCs of GARP knockdown. Mouse colon cancer MC38 cells were treated with MSC and siGARP‐MSC exosomes. Then, we found that MSC‐derived exosomes of GARP knockdown can inhibit cell proliferation, migration and invasion in mouse colon cancer MC38 cells. Furthermore, we also demonstrated that MSC‐derived exosomes of GARP knockdown can suppress IL‐6 and JAK1/STAT3 pathways. These results illuminate that GARP is a novel and promising target for MSC‐derived exosomes of gene therapy in the mouse colon cancer.

## 2 MATERIALS AND METHODS

### 2.1 Cells and cell culture

Mouse colon cancer MC38 cell line and mouse mesenchymal stromal cells were obtained from the Cell Bank Type Culture Collection of Chinese Academy of Sciences and cultured in RPMI‐1640 medium (Gibco, Invitrogen) containing 10% foetal bovine serum (FBS, Gibco) at 37°C in a humidified incubator containing 5% CO_2_.

### 2.2 siRNA transduction

The siRNAs of GARP (siGARP‐1, siGARP‐2 and siGARP‐3) and control siRNA (siNC) were purchased from GenePharma. Three siRNAs were transfected into MSCs using Lipofectamine RNAiMAX Reagent (Invitrogen), respectively, and named as siGARP‐MSC. Six hours after transfection, the cell culture medium was refreshed and cells were continued to be cultured. Cells were harvested for further assay 48 hours post‐transfection.

### 2.3 Isolation of exosomes

To extract exosomes, cells were cultured in medium containing exosome‐depleted FBS (System Biosciences). The exosome pellet was suspended in serum‐free medium. Exosomes were extracted from the supernatant of MSCs and GARP knockdown MSCs and named as MSC and siGARP‐MSC exosomes. The rest of the MSC or GARP knockdown MSC supernatants were used as controls. Exosome isolation was performed using MagCapture Exosome Isolation Kit PS (Wako), according to the manufacturer's protocol.

### 2.4 Cell treatment

MC38 cells were treated with MSC and siGARP‐MSC exosomes at a concentration of 10 μg/mL for 48 hours.^12^ Untreated MC38 cells were used as a control. After that, MC38 cells were collected for further study.

### 2.5 CCK‐8 assay

After cell counting, cells (5 × 10^3^ per well) were added to a 96‐well plate and then cultured for 48 hours. After 48 hours or culture, ten microlitres of CCK‐8 reagent (Beyotime) were added into each well and mixed gently. The cells were cultured for another hour under 37°C. Finally, the absorbance value was measured 450 nm by using a microplate luminometer reader (ELx808 Bio‐Tek Instruments).

### 2.6 Colony formation assay

Cells treated with MSC or MSC and siGARP exosomes were plated onto 6‐well plates at a density of 1 × 10^3^ or 3 × 10^3^ cells per well and continued to be cultured at 37°C and 5% CO_2_. After two weeks, the cells were fixed in 4% paraformaldehyde, stained with 1% crystal violet and then imaged and counted under a light microscope.

### 2.7 Wound‐healing assay

The horizontal lines were drawn at the bottom of 6‐well plates outside with sterilized rulers and markers. 1 × 10^5^ cells plated in the 6‐well plates were cultured for 24 hours, and perpendicular lines were drawn with the tip of the pipettor. Cells were cultured in the medium for another 24 hours, and the scratch wound was captured with microscope under 40‐fold magnification. And, the scratch area was counted and analysed by using ImageJ software.

### 2.8 Transwell invasion assay

For the invasion assays, chamber inserts were pre‐coated with BD Matrigel (BD Biosciences) and medium overnight under sterile conditions. Then, 1 × 10^5^ cells were seeded in the upper chamber. After 24 hours, cells on the top side of each insert were scraped off and the rest of the cells was fixed in methanol and stained using crystal violet. The cells of three random microscopic fields were counted per field for each group by using ImageJ, and these experiments were independently repeated at least three times.

### 2.9 Enzyme‐linked immunosorbent assay (ELISA)

The levels of interleukin‐6 (IL‐6), Janus Kinase 1 (JAK1), and signal transducers and activators of transcription three (STAT3) in the cell supernatant were determined using a commercial ELISA kit (BD Biosciences), according to the manufacturer's protocol.

### 2.10 RNA extraction and quantitative real‐time PCR

Total RNA was extracted using Eastep™ Universal RNA Extraction Kit (Promega), according to the manufacturer's handbook, and the concentration of total RNA was measured using a NanoDrop^®^ ND‐1000 (NanoDrop) at the wavelengths of 230, 260 and 280 nm. Complementary DNA was reversely transcripted using commercial iScript™ cDNA Synthesis Kit (Bio‐Rad), according to the manufacturer's instructions. A quantitative real‐time PCR assay was conducted using the SuperReal PreMix Plus (TianGen) and performed on an ABI 7,500 fast real‐time PCR system (ABI). The data were processed according to the 2^‐△△Ct^ method.

### 2.11 Western blot analysis

Cells were collected and lysed, and the proteins were extracted. The protein concentration was determined using a BCA kit. Before SDS‐PAGE, the same amount of loading buffer was added to the proteins to make a mixture, and the protein samples were boiled at 100°C for 10 minutes. Then, 10% SDS‐PAGE was performed and the proteins were transferred onto the PVDF membrane. Skim milk powder in PBST was used for blocking during 2 hours at 22‐25°C. The PVDF membrane was incubated with a primary antibody for 1 hour at 22‐25°C and then was washed three times with PBST for 10 minutes each time, including CD9 (Abcam, ab92726), CD63 (Abcam, ab217345), CD81 (Abcam, ab109201) and GARP (Abcam, ab231214). The PVDF membrane was incubated for 1 hour with the corresponding secondary antibody, and the PVDF membrane was washed three times with PBST. Protein bands were detected using an ECL detection kit (ECL; Thermo Scientific).

### 2.12 Statistical analysis

Statistical analysis was performed using SPSS 18.0 software (SPSS Inc) and GraphPad Prism Software version 6 (GraphPad Software, Inc). Student's *t* test was used to compare the control and treated samples. For all comparisons, differences were considered significant when *P* < .05.

## 3 RESULTS

### 3.1 The construction of GARP knockdown system in mouse MSC

**Figure 1:**
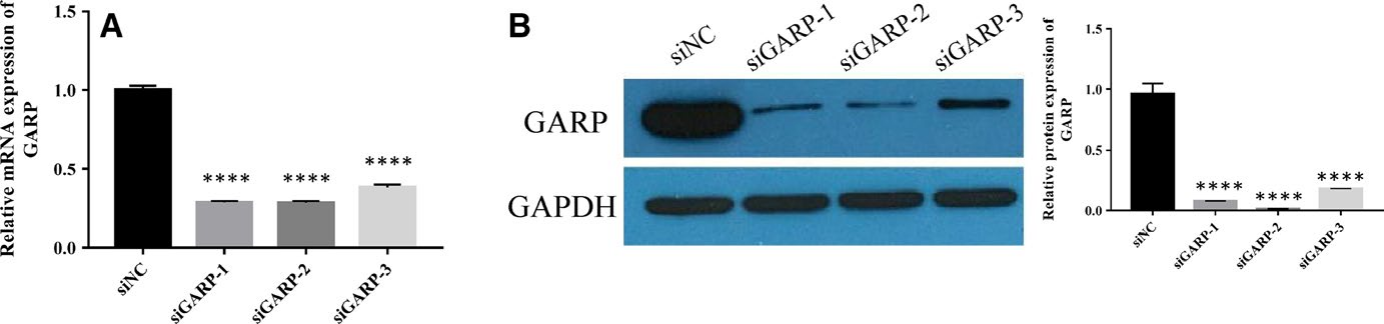
The construction of GARP knockdown system in mouse MSCs. Three kinds of siRNAs of GARP, including siGARP‐1, siGARP‐2 and siGARP‐3, were transfected into mouse MSCs to establish GARP knockdown system, and siNC was used as a control. A, The mRNA expression of GARP in the GARP knockdown system by using RT‐qPCR; B, the protein level of GARP in the GARP knockdown system by using Western blot analysis (****P < .0001 vs siNC)

To establish GARP knockdown system, the GARP siRNAs, including siGARP‐1, siGARP‐2 and siGARP‐3, were transfected into mouse MSCs and cells were collected for the verification of transcription and protein levels. As shown in Figure 1A, the mRNA level of GARP was significantly reduced in the siGARP‐1, siGARP‐2 and siGARP‐3 group compared with siNC group. Furthermore, the knockdown efficiency of siGARP‐2 was the best one among the siRNAs. Western blot analysis was performed to verify the protein level of GARP in the GARP knockdown system, and the results showed that GARP at the protein level obviously decreased in these siRNAs group, compared with siNC group (Figure 1B). The protein expression of GARP in the siGARP‐2 was the lowest, indicating that the knockdown efficiency of siGARP‐2 was the best one among these groups. Based on these results, the knockdown system of GARP in MSCs was successfully constructed.

### 3.2 Extraction and identification of exosomes from MSCs and siGARP‐MSCs

**Figure 2:**
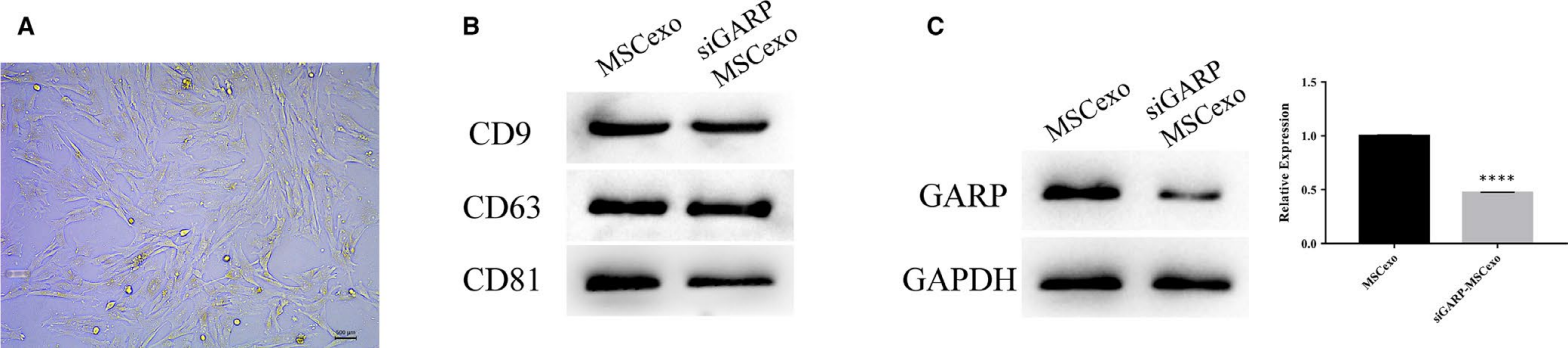
Extraction and identification of exosomes from MSCs and siGARP‐MSCs. A, The morphology of MSCs; B, the marker proteins of exosomes surface, including CD9, CD63 and CD81, from the precipitation of MSCs, the supernatant of MSCs and the supernatant of GARP knockdown MSCs, were measured by using Western blot analysis; C, the protein level of GARP from MSC and siGARP‐MSC exosomes was evaluated by using Western blotting. MSCexo = 'MSC exosome'; siGARP‐MSCexo = 'siGARP‐MSC exosome' (****P < .0001 vs MSCexo)

We found that the morphology of MSCs was uniform under the electron microscope and that their growth state was good (Figure 2A). Then, the exosomes were extracted from MSCs using MagCapture Exosome Isolation Kit PS (Wako) and the surface marker proteins of exosomes were identified by using Western blot. Figure 2B showed that the CD9, CD63 and CD81 proteins of MSCs or siGARP‐MSC exosomes were measured using the corresponding primary antibody, and the precipitation of MSCs or siGARP‐MSCs showed a negative result of exosome surface marker proteins, suggesting that MSC exosomes were successfully isolated. Similarly, siGARP exosomes were extracted by using the same exosome isolation kit and this isolation was verified to be successful based on the confirmation of exosome surface marker proteins CD9, CD63 and CD81 (Figure 2B). To verify the GARP expression of MSC and siGARP‐MSC exosomes, Western blot was performed and the results showed that the GARP protein level of siGARP‐MSC exosomes was significantly impaired, compared with that of MSC exosomes (Figure 2C).

### 3.3 Exosomes of siGARP‐MSC inhibited cell proliferation, migration and invasion in MC38 cells

**Figure 3:**
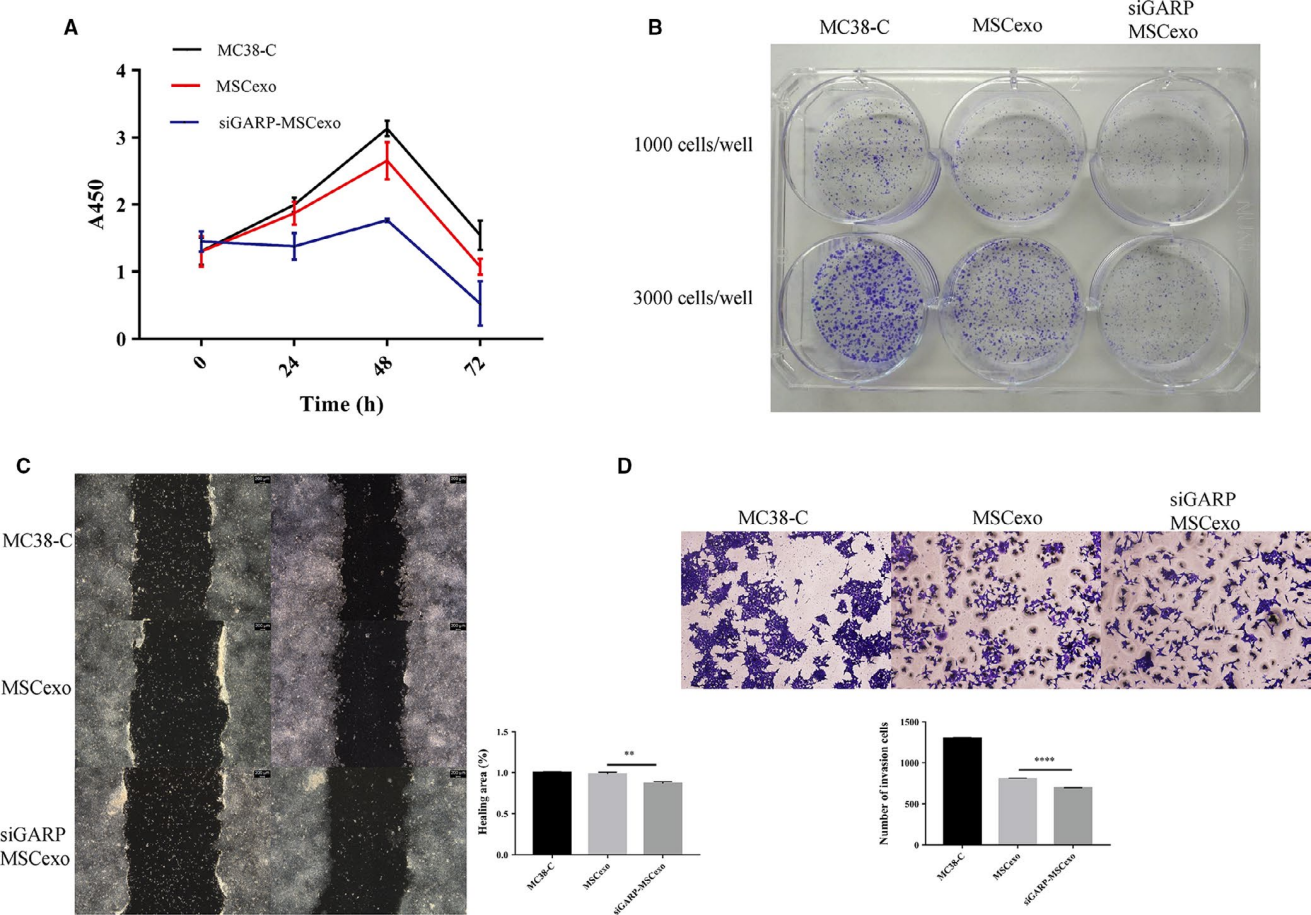
Exosomes of siGARP‐MSCs inhibited cell proliferation, migration and invasion in MC38 cells. MC38 cells were treated with siGARP‐MSC or MSC exosomes, and non‐treated MC38 cells were used as a control. A, cell viability of MC38 cells in these three groups; B, colony formation ability of MC38 cells from these three treatments; C, cell migration ability of MC38 cells from these three groups; D, cell invasion ability of MC38 cells from these three groups. MC38‐C = control (**P < .01, ****P < .0001 vs MSC38‐C)

In order to explore the role of siGARP‐MSC exosomes in cancer treatment, mouse colon cancer MC38 cells were treated with siGARP‐MSC and MSC exosomes and untreated MC38 cells were used as a control. Then, cell proliferation, migration and invasion ability of the MC38 cells treated with different exosomes were evaluated, respectively. Through CCK‐8 and colony formation assays, we found that cell proliferation ability of cells treated with different exosomes was impeded, compared with the control. Importantly, the cell proliferation ability of cells treated with siGARP‐MSC exosomes was lower than that of cells treated with MSC exosomes (Figure 3A,B). Moreover, cell migration was also determined, and the results suggested that the cell migration ability of cells treated with MSC or siGARP‐MSC exosomes was reduced, compared with untreated MC38 cells. Similarly, siGARP‐MSC exosomes indicated an inhibitor effect on cell migration ability, compared with MSC exosomes (Figure 3C). At the same time, MC38 cells treated with exosomes showed that cell invasion was also inhibited in the MC38 cells treated with MSC or siGARP‐MSC exosomes (Figure 3D), and cell invasion ability was the lowest in the MC38 cells treated both with siGARP‐MSC exosomes.

From these results, exosomes of siGARP‐MSC probably inactivated cell biological function, including cell proliferation and invasion, of MC38 cells.

### 3.4 Exosomes of siGARP‐MSC impaired IL‐6 and JAK1/STAT3 signalling pathways

**Figure 4:**
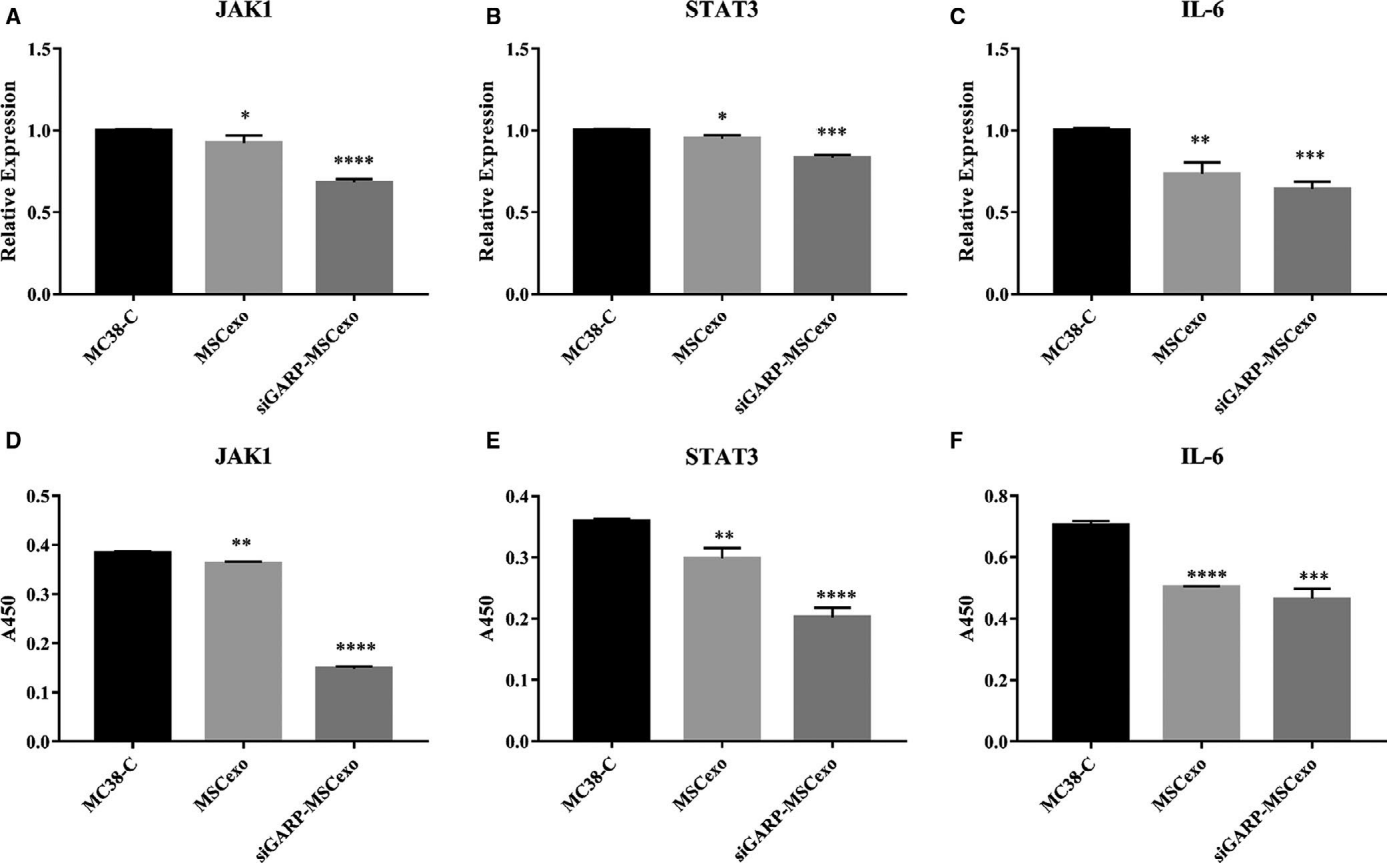
Exosomes of siGARP‐MSC impaired JAK1/STAT3 signalling pathway and IL‐6. The mRNA expression of IL‐6, JAK1 and STAT3 was measured using RT‐qPCR, and the content of IL‐6, JAK1 and STAT3 was quantified using ELISA. A, The mRNA level of JAK1; B, STAT3; C, and IL‐6; D, the content of JAK1; E, STAT3; F and IL‐6 in the supernatant of MC38 cells. MC38‐C = 'control'; MSCexo = 'MSC exosome'; siGARP‐MSCexo = 'siGARP‐MSC exosome' (*P < .05, **P < .01, ***P < .001, ****P < .0001 vs control)

Subsequently, we wondered whether IL‐6 and JAK/STAT signalling pathways were inhibited when cell migration and invasion abilities were impaired by siGARP‐MSC exosomes. Therefore, the mRNA level of IL‐6, JAK1 and STAT3 was measured by using qRT‐PCR. As we expected, the expression of JAK1, STAT3 and IL‐6 at the mRNA level was greatly reduced in MC38 cells treated with siGARP‐MSC exosomes, compared with the control and MSC exosomes (Figure 4A‐C). Furthermore, the supernatant content of JAK1, STAT3 and IL‐6 was also verified by using ELISA and the tendency of JAK1, STAT3 and IL‐6 was consistent with their mRNA expression (Figure 4D‐F).

## 4 DISCUSSION

Mesenchymal stem cells (MSCs), also referred to as mesenchymal stromal cells, are adult stem cells capable of self‐renewal and multilineage differentiation. MSCs were first discovered from bone marrow and reported approximately 40 years ago by Friedenstein and his coworkers.^13^ Later studies have suggested that MSCs exist in a variety of organs, including the brain, liver, lungs, kidneys, muscles, thymus, pancreas, skin and bone marrow adipose tissue.^14^ Therefore, a large number of MSC‐derived exosomes were isolated from different MSCs, as mentioned above.

MSC‐derived exosomes can promote and inhibit tumorigenesis. MSC‐derived exosomes that inhibit tumorigenesis can be considered targeted therapies for tumours. Adipose MSC‐derived exosomes were suggested to inhibit prostate cancer via delivery of miR‐145 by reducing the activity of Bcl‐xL and promoting apoptosis through the caspase‐3/7 pathway.^15^ According to Wu *et al*, extracellular vesicles (EVs) from human umbilical cord Wharton's jelly MSCs reversed the development of bladder carcinoma cells, possibly by down‐regulating phosphorylation of Akt protein kinase and up‐regulating cleaved caspase‐3.^16^ It has been reported that MSCs induced the invasiveness of breast cancer cells, partly through MSC‐derived exosomes.^17^ Therefore, in the present study, we wanted to explore the effect of MSC‐derived exosomes on mouse colon cancer MC38 cells.

Interestingly, plenty of studies have reported an amplification of the GARP gene in tumours, especially in those with an invasion or metastatic potential, suggesting a role of this gene product in regulating aggressive tumour biology, once deregulated.^18^, ^19^, ^20^ Rachidi et al found that targeting platelets improved adoptive T‐cell therapy of multiple cancers in mice. Transforming growth factor‐β (TGF‐β) from platelets decreased T‐cell function, largely through the expression of the TGF‐β docking receptor GARP.^21^ Therefore, we deduced that GARP was a target in cancer therapy. In this study, MSC‐derived exosomes targeting GARP gene were used to treat MC38 cells. At first, GARP knockdown system in MSCs was established with three kinds of GARP siRNAs and we found that the knockdown efficiency of siGARP‐2 was the best one among them based on the mRNA and protein levels of GARP (Figure 1). Subsequently, exosomes were isolated from MSC and siGARP‐MSCs, and these two types of exosomes were identified by the marker proteins, including CD9, CD63 and CD81. In Figure 2B, protein markers including CD9, CD63 and CD81 exist in the MSC and siGARP‐MSC exosomes, corroborating the successful isolation of MSC and siGARP‐MSC exosomes. We further verified the protein expression of GARP in the MSC and siGARP‐MSC exosomes, and the results showed that the protein level of GARP in siGARP‐MSC exosomes was significantly reduced compared with that of MSC exosomes (Figure 2C).

Metelli and his team have reported that the up‐regulation of GARP in murine mammary cancer cells promoted TGF‐β activation, tumour growth and metastasis.^10^ In the present study, siGARP‐MSC exosomes were used to treat MC38 cells and MSC exosome treatment was used as a control. Then, cell proliferation, migration and invasion were measured. In Figure 3, it is possible to observe that siGARP‐MSC exosomes suppressed cell proliferation, migration and invasion of MC38 cells, suggesting that knockdown of GARP impeded tumour growth and metastasis. Our results were consistent with those of Metelli.

Glycoprotein A repetition predominant has emerged as a critical regulator of latent TGF‐β activation.^10^ By binding to latent TGF‐β, GARP acts as a docking receptor that concentrates latent TGF‐β on the cell surface and enhances its final activation.^22^ Tang et al demonstrated that TGF‐β directly activates the JAK1/STAT3 axis to induce hepatic fibrosis in coordination with the SMAD pathway.^23^ In addition, Kuhn et al reported that signal transducer and activator of transcription 3 (STAT3) was another transcription factor that regulated GARP gene expression and interleukin (IL)‐6 administration to CD4^+^ naïve T cells, which was sufficient to restrain GARP transcription and expression via the STAT3 signalling pathway.^6^ The JAK1/STAT3 axis and IL‐6 were studied in MC38 cells treated with siGARP‐MSC and MSC exosomes, and the results suggested that siGARP‐MSC exosomes inhibited IL‐6 secretion and inactivated JAK1/STAT3 axis (Figure 4), which was consistent with the results of Tang.^23^ However, in the present study, the effect of the exosomes of siGARP‐MSC on cell proliferation and invasion was only studied in MC38 cells, and the role of the exosomes of siGARP‐MSC in other mouse colon cancer cell lines will be studied in the future.

In summary, GARP knockdown system was constructed using MSCs and exosomes were isolated from both MSC and siGARP‐MSCs, respectively. Then, the protein markers in the surface of exosomes, including CD9, CD63 and CD81, were verified in these two types of exosomes and the protein level of GARP in the siGARP‐MSC exosomes was confirmed. Furthermore, siGARP‐MSC exosomes were used to treat MC38 cells and MSC exosomes were used as a control, and we found that siGARP‐MSC exosome treatment inhibited cell proliferation, migration and invasion. At the same time, we demonstrated that siGARP‐MSC exosome treatment impedes IL‐6 secretion and JAK1/STAT3 pathway. In this study, the effect of siGARP‐MSC exosomes on cell proliferation and invasion of MC38 cells was elucidated, and MSC‐derived exosomes targeting GARP could be used as a novel cancer therapy strategy for mouse colon cancer.

## CONFLICT OF INTEREST

The authors confirm that there are no conflicts of interest.

## AUTHOR CONTRIBUTION

**Haizhou Xing:** Conceptualization (lead); Funding acquisition (lead); Investigation (lead); Methodology (equal); Project administration (lead); Supervision (lead); Validation (lead); Writing‐review & editing (equal). **Chunyan Liang:** Data curation (equal); Formal analysis (equal); Investigation (equal); Methodology (supporting); Resources (supporting); Software (equal); Visualization (equal); Writing‐original draft (lead). **Xintong Xu:** Data curation (equal); Formal analysis (equal); Investigation (supporting); Methodology (supporting); Software (equal); Visualization (equal). **Hui Sun:** Investigation (supporting); Methodology (supporting); Resources (lead); Visualization (equal). **Xiaojun Ma:** Conceptualization (equal); Investigation (equal); Methodology (supporting); Supervision (equal); Validation (supporting); Writing‐original draft (supporting).**Zhongxing Jiang:** Investigation (supporting); Methodology (equal); Resources (equal); Validation (supporting).

## Data Availability

Research data are not shared.
